# Supplementary material for: Heat stress reveals a fertility debt owing to postcopulatory sexual selection
Source: Evol Lett. 2023 Mar 16;8(1):101–13. doi: 10.1093/evlett/qrad007 (PMC10872150; doi:10.1093/evlett/qrad007)
Supplement: qrad007_suppl_Supplementary_Material [file qrad007_suppl_supplementary_material.pdf]

Supplementary note S1:

A heuristic model of postcopulatory sexual selection and environmental sensitivity of fertility

We modelled the environmental sensitivity of fertility arising from reproduction-maintenance trade-offs in the germline by employing a life-history framework and a version of the "Y-model" of resource acquisition and allocation[1,2]. The aim was to provide general and qualitative predictions for how a long history of strong postcopulatory sexual selection would affect male fertility under abrupt environmental change. We therefore constructed a very simple model with as few assumptions as possible. We did not consider trade-offs with somatic performance or trade-offs between current and future reproduction. Moreover, as the focus was on predicting how evolutionary history would affect immediate responses to future climate change (e.g. heat waves), we did not model adaptive plasticity in germline allocation (optimal strategies were assumed to be fixed), or further evolution following the environmental change.

Individual condition (C) was assumed to determine the amount of resources that can be allocated to germline maintenance (M) in form of anti-oxidative defence and repair needed to maintain gamete viability[3,4], or reproductive effort (R) in form of production of gametes and ejaculatory components that increase a male's success in sperm competition[5,6], such that:

$$C = R + M; \quad R = kC; \quad M = (1-k)C. \qquad \textbf{Eq. 1}$$

where k is the proportion of resources allocated to reproduction. Gamete viability, ζ, is assumed to be dependent on the amount of maintenance per reproductive effort (i.e. per gamete or ejaculate volume):

$$\zeta \approx \left[\frac{(1-k)}{(1+k)}\right]^{a} \qquad \textbf{Eq. 2}$$

where a describes environmentally dependent consequences of sub-maximal germline maintenance, such that some environmental conditions will impair fertility more than others (e.g. hot temperature or high salinity increases a). The addition of +1 to the denominator of Eq. 2 assures that viability ranges between 0 and 1, but we note that this choice was arbitrary and that other expressions for Eq. 2 resulted in the same qualitative results. While we here express the effect of germline maintenance (M) on gamete viability, we note that gamete quality is directly related to the survivorship and quality of offspring in species with limited parental care, and hence, our results relate directly to measures of offspring survival (as measured in the empirical data). If reproductive success follows a power function of reproductive effort, and if fitness, ω, is the product of sperm competition success and gamete viability, then:

$$\omega \approx R^b \zeta = (kC)^b \left[\frac{(1-k)}{(1+k)}\right]^a \qquad \textbf{Eq.3}$$

where parameter b describes how reproductive effort translates into postcopulatory reproductive success. When $b = 1$, success in sperm competition is directly proportional to the amount of resources invested, as envisioned in "fair raffle" models of sperm allocation under risk of sperm competition[7]. When $b < 1$, reproductive success is less than proportional to investment and postcopulatory sexual selection is relatively weak as expected when risk of sperm competition is low, whereas $b > 1$ gives a disproportionate advantage to individuals investing more in reproductive effort and sexual selection is very strong. We note that this last scenario implies some sort of threshold mechanism at play, where male allocation need to exceed some particular value to achieve fertilization. Such mechanisms could, for example, be at play through female choice of male sperm, but we note that empirical evidence for this hypothesis is scarce[8]. Individuals in a population experiencing strong sexual selection (b>1) need to invest much more in reproductive effort to secure a significant share of paternity relative to individuals from a population where sexual selection is weak (b<1). Given a trade-off between investment in reproductive effort and germline maintenance (Eq. 1), such excessive germline allocation is predicted to result in reduced fertility, an effect that is particularly pronounced under harsh environmental conditions (Fig. S1.1).

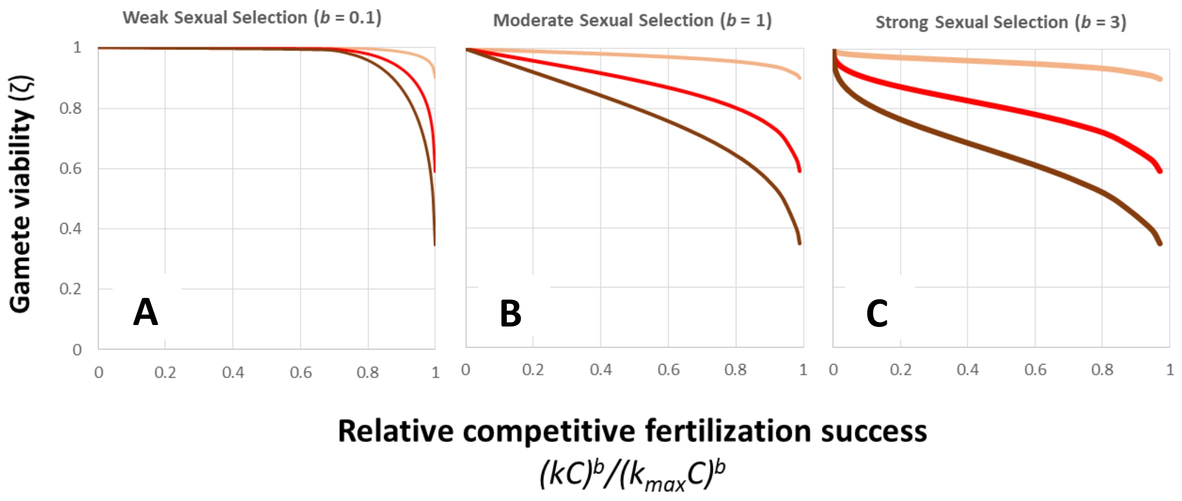

Supplementary Figure S1.1. The relationship between competitive reproductive success (i.e. success in sperm competition) and gamete viability for different strengths of sexual selection (b = 0.1; 1; 3 from panel **A-C**) and viability selection (a; salmon = 0.02, red = 0.10, brown = 0.20). To facilitate interpretation, competitive reproductive success is expressed relative to the success of an individual with the same condition (C) investing all resources in reproduction (k_{max} = 1). Optimal allocation in each scenario is found by maximizing the product of gamete viability and competitive fertilization success, corresponding to the area under each curve's inflection point. Individuals experiencing strong sexual selection (panel **C**) need to invest more in reproduction to get the same share of paternity compared to individuals experiencing weak sexual selection (panel **A**) and pay a fertility cost in terms of reduced gamete viability. This cost becomes more pronounced in harsh environments (brown lines).

The optimal germline allocation strategy (k_{opt}) for different strengths of sexual selection (*b*) and viability selection (*a*) is given by differentiation of equation (3) with respect to *k*:

$$k_{opt} = \frac{\sqrt{b^2+a^2}-a}{b} \qquad \textbf{Eq. 4}$$

which shows that optimal allocation to reproduction, k_{opt}, increases with the strength of sexual selection, b, and decreases with environmentally dependent viability selection, a. Unsurprisingly, increased sexual selection does indeed lead to decreased gamete viability as follows from the trade-off scenario described by equation (1) (Supplementary Fig. S1.1 and Fig. S1.2A). Optimal allocation (and resulting fertility) is independent of condition, C, when reproductive success is a power function of investment, but we note that reproductive effort and gamete viability can either be increasing or decreasing functions of condition, depending on the fitness functions used (results not shown, but see: [8–10]).

What consequences do differences in mating system and a history of intense sperm competition (high b) have for fertility responses to increased environmental stress (increases in a)? We illustrate these effects by first replacing a in equation (4) with a_{anc}, representing viability selection in a relatively benign ancestral environment, making it possible to solve for k_{opt} for different values of b. We then replace k in equation (2) with this expression for k_{opt} and differentiate with respect to a to show how gamete viability, ζ, is affected by increasing environmental stress for allocation strategies that have evolved under different scenarios of sexual selection and viability selection in the ancestral environment:

$$\frac{d\zeta}{da} = \left[\frac{\sqrt{b^2+a_{anc}^2}-b}{a_{anc}}\right]^a . \ln\left[\frac{\sqrt{b^2+a_{anc}^2}-b}{a_{anc}}\right] \qquad \textbf{Eq. 5}$$

Predictions from equation (5) are presented in Supplementary Fig. S1.2B (see also Fig. 1B in main text) and show that, for any strength of viability selection in the ancestral environment, populations that have evolved under a history of strong sexual selection are predicted to suffer a greater fertility loss following increased environmental stress.

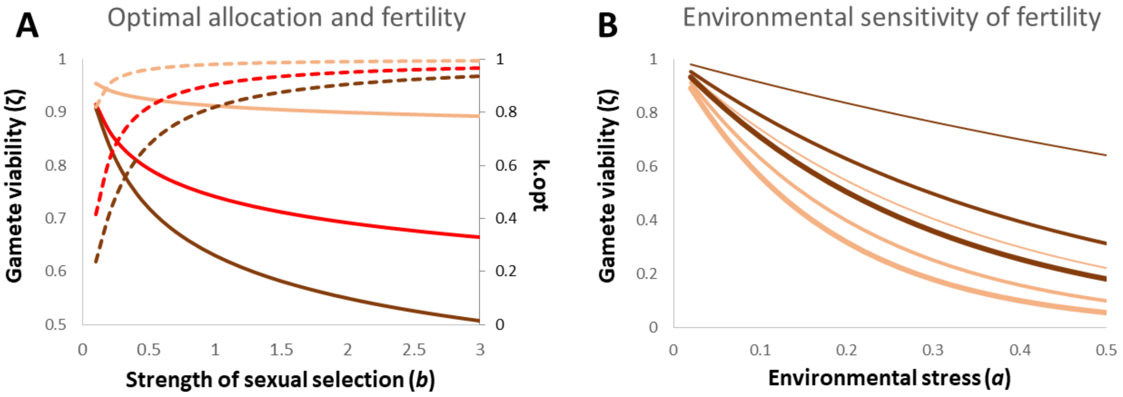

Supplementary Figure S1.2. In **A**) the optimal reproductive effort (k_{opt}; hatched lines) and resulting gamete viability (ζ; full lines) for different levels of sexual selection (*b*) and viability selection (*a*; salmon = 0.02, red = 0.10, brown = 0.20). In **B**) the change in gamete viability as environmental stress (*a*) changes from ancestral conditions (a_{anc}; salmon = 0.02, brown = 0.20) for populations that have evolved optimal allocation under either weak (*b* = 0.1, thin lines), strong (*b* = 1, intermediate lines) or very strong (*b* = 3, thick lines) postcopulatory sexual selection.

Extended discussion of model assumptions and the relation between predictions and data

Our aim with the described model was to generate predictions for how previous differences in mating system and adaptations to postcopulatory sexual selection would affect immediate responses to environmental stress, qualitative predictions that could be tested with our empirical data. In our simple model we therefore described viability selection and sexual selection by power functions with exponents *a* and *b* without discussing mechanisms behind exactly what determines *a* and *b.* Indeed, we made the very simple assumption that sperm competition should be stronger (*b* greater) in a population where there is multiple mating and mate choice (the N+S and S regimes) compared to a population where there is no sexual selection (the N regime). This simple logic is supported by both theoretical models on sperm and ejaculate allocation[8] and a rich empirical literature[5,8,11,12]. We also made the reasonable assumption that viability selection increases (via exponent *a*) at more stressful conditions, but we note that the use of power functions to describe both these relationships were arbitrarily chosen.

Indeed, sperm competition is the results of multifaceted frequency-dependent pre- and postcopulatory processes[8,12]. Nevertheless, even though we did not explicitly model frequency-dependence here but rather manipulated (i.e. fixed) the strength of sexual selection at different values of *b* to evaluate effects on fertility under environmental change, ESS models on sperm competition reassuringly produce the same qualitative results as we obtained here with reference to how the risk of sperm competition benefits increased allocation to ejaculate traits that increase competitive reproductive success[8] (parameter *k* in our model). For example, in Parker's (1990) simple "fair raffle" scenario[7], *k* is directly proportional to the risk of sperm competition. Further note that the risk of sperm competition in our N regime is = 0, and essentially ~1 in our N+S and S lines (based on remating rates estimated in[13]). Hence, both our model and ESS models (reviewed in: [8]) make the qualitative

prediction that allocation to *k* should be greater in N+S and S lines relative to N lines. Our model then increased environmental stress (*a*) to show that this allocation decision results in compromised fertility in the N+S and S regimes under the assumption of a trade-off between gamete viability and sperm competition success. Moreover, because the optimal allocation decision is determined by a balance between viability selection and sexual selection, the model predicts that the S regime, where females unconditionally contributed only two offspring in each generation (and viability selection therefore was weaker compared to the N and N+S regime), should show the greatest environmental sensitivity. We did indeed find evidence for these qualitative predictions in our data (see main text).

The model predicts that there should still be differences in fertility between our evolution regimes in benign settings as long as there is some viability selection ($a > 0$) , although these differences should be much smaller than at high environmental stress (*a* increases). We do not see a difference in fertility at benign temperature in our empirical data. We believe a very likely reason for this is that these lines also evolve other components related to fertility. For example, we have found evidence that N+S lines are overall in better condition and have higher fecundity, suggesting that the overall stronger selection in this regime may have more efficiently purged deleterious alleles[14]. Another (not mutually exclusive) explanation is that environmental harshness in our experiment (exponent *a* in the model) is close to 0 in our benign conditions (corresponding to the scenario with $a = 0.02$ in figures S1.1 and S1.2). Given the ad libitum egg-laying substrate and high fertility of *C. maculatus* in laboratory conditions (egg-to-adult survival typically at 95%, where the 5% mortality could be due to handling), suggest that *a* is indeed close to 0. If so, the model predicts very small (statistically undetectable) difference in fertility between regimes when held at benign conditions. Moreover, even though our experimental evolution lines have been propagated for a relatively long time compared to other similar laboratory evolution experiments manipulating mating system, we expect them to not have reached their new evolutionary optima (allocation decisions maximizing fitness in their respective lab environments). Hence, there should also be some caution when comparing empirical data and model predictions.

Finally, because (postcopulatory) sexual selection is inherently a frequency dependent process, it is likely that abrupt changes in the environment (increases in *a*) could themselves modulate parameter *b*, by for example changing the density and quality of rivalling males at mating sites[7,8,14–16]. If we are concerned with predicting how germline plasticity and future evolution of reproductive strategies in changing environments affect fertility and population health, such dynamics would ultimately need to be considered in more sophisticated mechanistic models incorporating frequency-dependent selection. Here, however, we kept *b* constant to describe the strength of sexual selection in the ancestral environment as we were interested in generating predictions for immediate fertility responses under abrupt environmental change attributed to the organism's evolutionary history of natural and sexual selection. We then could compare these qualitative predictions directly with our empirical data (see main text).

References:

Houle, D. Genetic Covariance of Fitness Correlates: What Genetic Correlations are Made of and Why it Matters. Evolution 45, 630 (1991). https://doi.org/10.1111/j.1558-5646.1991.tb04334.x

Jong, G. de & Noordwijk, A. J. van. Acquisition and Allocation of Resources: Genetic (CO) Variances, Selection, and Life Histories. American Naturalist. 139, 749–770 (1992). https://doi.org/10.1086/285356

Friedberg, E. C., Walker, G. C., Siede, W. & Wood, R. D. DNA Repair and Mutagenesis. (American Society for Microbiology Press, 2005).

Dowling, D. K. & Simmons, L. W. Reactive oxygen species as universal constraints in life-history evolution. Proceedings of the Royal Society of London. Series B: Biological Sciences. 276, 1737–1745 (2009). https://doi.org/10.1098/rspb.2008.1791

Snook, R. R. Sperm in competition: not playing by the numbers. *Trends Ecol. Evol.* 20, 46–53 (2005). https://doi.org/ 10.1016/j.tree.2004.10.011

Cook, P. A. & Wedell, N. Non-fertile sperm delay female remating. *Nature* 397, 486–486 (1999). https://doi.org/10.1038/17257

Parker, G. A. Sperm competition games: raffles and roles. *Proc. R. Soc. Lond. B Biol. Sci.* 242, 120–126 (1990). https://doi.org/10.1098/rspb.1990.0114

Parker, G. A. & Pizzari, T. Sperm competition and ejaculate economics. Biological Reviews. 85, 897–934 (2010). https://doi.org/10.1111/j.1469-185X.2010.00140.x

Kokko, H. Fisherian and “good genes” benefits of mate choice: how (not) to distinguish between them. Ecology Letters. 4: 322-326 (2001). https://doi.org/10.1046/j.1461-0248.2001.00224.x

Bonduriansky, R. & Day, T. The evolution of static allometry in sexually selected traits. Evolution 57, 2450–2458 (2003). https://doi.org/10.1111/j.0014-3820.2003.tb01490.x

Andersson, M. *Sexual Selection*. (Princeton University Press, 1994).

Arnqvist, G. & Rowe, L. *Sexual Conflict*. (Princeton University Press, 2005).

Baur, J. & Berger, D. Experimental evidence for effects of sexual selection on condition-dependent mutation rates. *Nat. Ecol. Evol.* 4, 737–744 (2020). https://doi.org/10.1038/s41559-020-1140-7

Martinossi-Allibert Ivain, Rueffler Claus, Arnqvist Göran, & Berger David. The efficacy of good genes sexual selection under environmental change. Proceedings of the Royal Society of London. Series B: Biological Sciences. 286, 20182313 (2019). https://doi.org/10.1098/rspb.2018.2313

Miller, C. W. & Svensson, E. I. Sexual Selection in Complex Environments. Annual Review of Entomology. 59, 427–445 (2014). https://doi.org/10.1146/annurev-ento-011613-162044

Svensson, E. I. & Connallon, T. How frequency-dependent selection affects population fitness, maladaptation and evolutionary rescue. Evolutionary Applications 12: 1243-1258. https://doi.org/10.1111/eva.12714

Supplementary figure S2:

Experimental design: main experiment

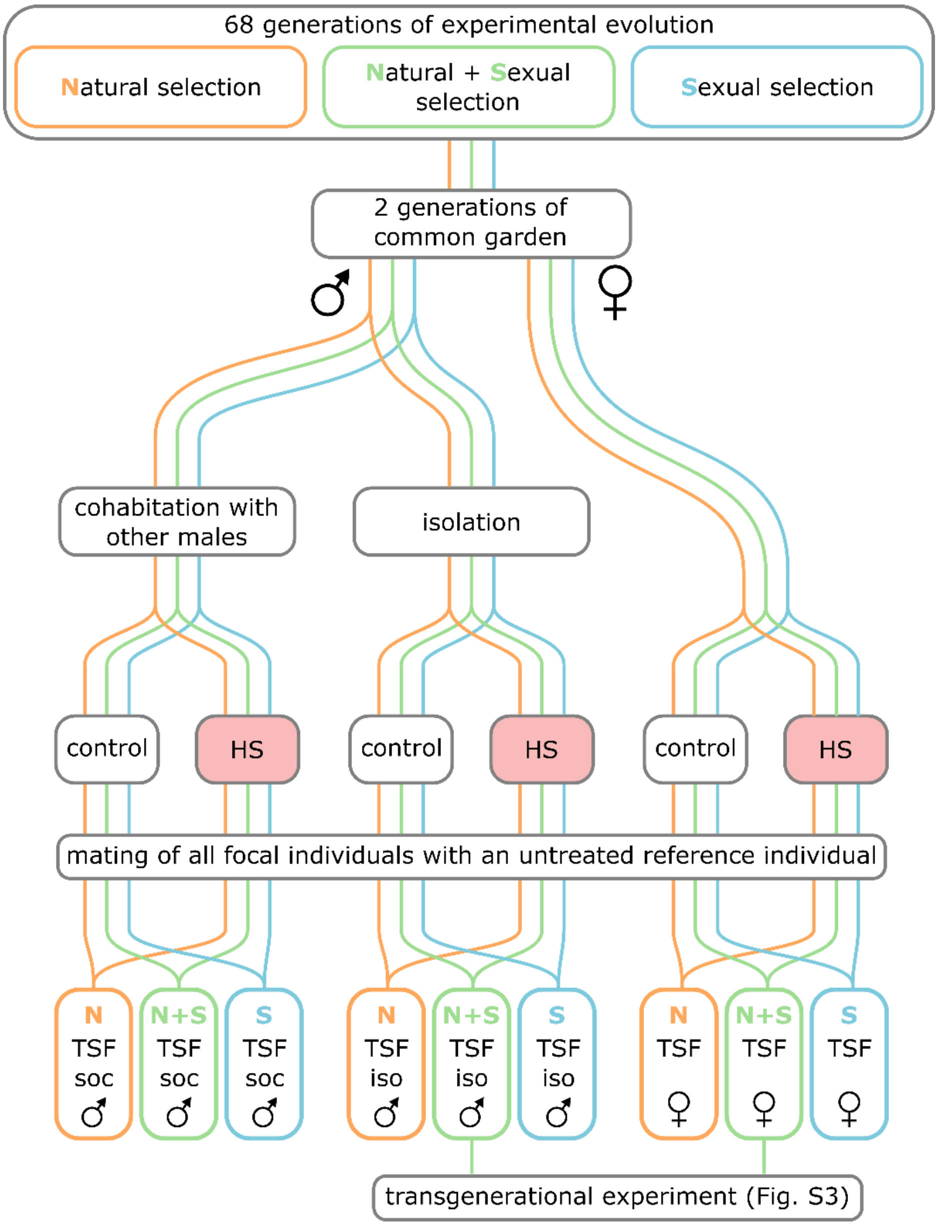

Design of the main experiment, including experimental evolution under the three alternative selection regimes, and two generations of common garden prior to the experimental generation. In the first experimental generation (parental), virgin beetles of all three regimes were picked within 24 hours after eclosion and males were either kept isolated or kept together with other males. Subsequently, half of the beetles of each regime and treatment combination were exposed to a heat shock. The thermal sensitivity of fertility was assessed by comparing heat shocked and control beetles within regime and treatment according to: TSF = 1 – ($offspring_{heat\ shocked}$ / $offspring_{control}$). F1 offspring of heat shocked and control N+S males and females were used for the assessment of transgenerational effects of heat shock (see supplementary figure S4).

Details of the experimental design used to manipulate mating system

The selection regimes used here were designed to manipulate the relative strength of selection on males and females. However, the way the regimes were implemented also caused small differences in the timing of propagation, the amount of egg-laying substrate per female, and container size during propagation, which we discuss below.

Timing of reproduction: The regimes impose slightly different reproductive schedules. In N + S lines males and females can mate and lay eggs for 48 hours after their emergence, in N lines males and females can mate for five hours after which females get 48 hours for egg-laying. Hence, the 48-hour egg-laying period is delayed by 5 hours in N compared to N+ S females. It has been shown that female *C. maculatus* age substantially faster once egg-laying starts (e.g., Tatar *et al.* 1993, Wilson 1994, Maklakov & Bonduriansky 2009). We therefore argue that this 5-hour shift is negligible, even in a short-lived species such as *C. Maculatus*, since the time period is very short and females have no access to beans, are not ovipositing during that time, and are kept at very low density (alone together with a single male). For females evolving under the S regime egg-laying is delayed by 48 hours compared to the N + S regime. However, in the S regime females are under relaxed selection (females contribute one female and one male offspring to the next generation) and females typically live around seven days while reproducing under crowded conditions. Thus, rather than imposing selection on the reproductive schedule of females, this regime removes selection on females all together.

Juvenile density: The number of beans per female varies slightly between the experimental evolution regimes (N + S: ~32 beans/female, N: ~39 beans/female, S: ~30 beans/female) (see Martinossi-Allibert *et al.* 2019, for details regarding population size under the various regimes). However, a single bean can sustain the development of more than 10 beetles and a female rarely lays more than 100 eggs.

If these differences in rearing conditions were of importance, we would expect the regimes to evolve differences in longevity and body size. We have found no such differences in adult body mass at generation 16 (Martinossi-Allibert *et al.*, 2019), or at generation 40 (White S, Bolund E and Berger D, unpublished data). The last experiment was designed to test for the evolution of sex-specific genetic (co)variances in life history, and sample size were therefore sizeable. While there was a slight tendency for differences in longevity between the three regimes in this experiment, it was polygamy that tended to have longer life (although marginally non-significant). Importantly, the polygamy regime was intermediate in our current experiment on thermal sensitivity (TSF), and any potential differences in longevity thus seem unlinked to our current results.

Use of different containers: To apply different selection pressures in the various experimental evolution regimes, we kept beetles in different containers and environments during the reproduction and egg-laying (N + S: mating and egg-laying in a large jar with beans, N: mating in a 60mm Petri-dish and egg-laying in a large jar with beans, S: mating in a large jar with a cardboard structure and egg-laying in a 60mm Petri-dish). These environments inevitably cause the beetles to receive different environmental cues depending on their selection regime. We do, however, believe that we designed a selection protocol that minimises these differences. The Petri-dish used for mating in the N regime was applied for a short time (5h) and the main objective with this treatment was to allow females to mate while removing sexual selection and conflict. When the Petri-dish treatment is applied in the S regime, it is after mating interactions, to females that only contribute 2 offspring to the next generation (effectively removing fecundity selection), so the applied Petri-dish treatment is extremely unlikely to have enforced any selection in the S regime. What is important in this species is the density and complexity of the environment during mating interactions, hence the card-board structure placed in the S-treatment.

References:

Maklakov, A., Bonduriansky, R. (2009) Sex differences in survival cost of homosexual and heterosexual interactions: evidence from a fly and a beetle. *Anim. Behav.* 6: 1375-1379. https://doi.org/10.1016/j.anbehav.2009.03.005

Martinossi-Allibert, I., Thilliez, E., Arnqvist, G., & Berger, D. (2019). Sexual selection, environmental robustness, and evolutionary demography of maladapted populations: A test using experimental evolution in seed beetles. *Evolutionary Applications*, *12*(7), 1371–1384. https://doi.org/10.1111/eva.12758

Tatar, M., Carey, J.R. & Vaupel, J.W. (1993) Long-term cost of reproduction with and without accelerated senescence in *Callosobruchus maculatus*: analysis of age-specific mortality. *Evolution* 47: 1302– 1312. https://doi.org/10.1111/j.1558-5646.1993.tb02156.x

Wilson, K. 1994. Evolution of clutch size in insects. II. A test of static optimality models using the beetle *Callosobruchus maculatus* (Coleoptera: Bruchidae). *J. Evol. Biol.* 7: 365– 386. https://doi.org/10.1046/j.1420-9101.1994.7030365.x

Supplementary figure S3:

Daily temperature curve for Lomé, Togo

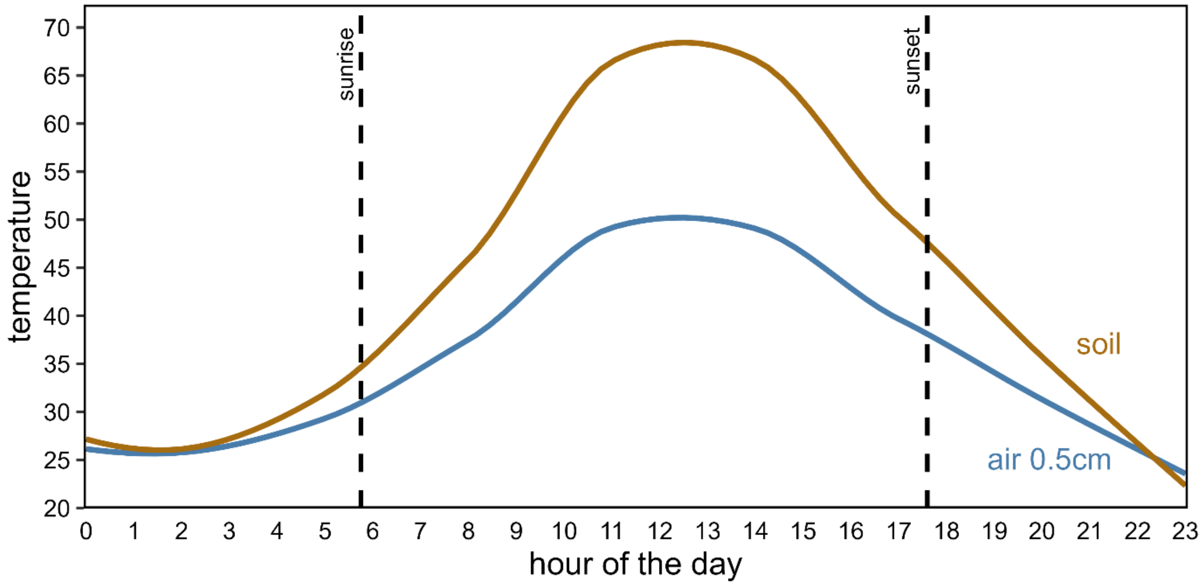

Soil and air temperature curve as it may occur on a day in November, the hottest month of the year, in Lomé, Togo (06°10#N 01°13#E), the original collection site of the beetle stock used here. The presented temperatures have been estimated using NicheMapR (version 3.2.0; Kearney and Porter, 2016, *Ecography*), an R software package interpolating climate data in order to model microclimates on a very fine scale. The shown curves represent soil temperature and air temperature in a height of 0.5cm. We assumed a climate change scenario of global warming of 1.5°C, a day with no cloud coverage, even ground, average local wind speed, 15% shaded area, and conservative values for soil albedo (15%) and emissivity (0.9).

Supplementary table S4:

Sample sizes

Table S2.1: Sample size per cell for analysis of male thermal sensitivity of fertility

Regime	Ctrl, iso	HS, iso, 1st	HS, iso, 2nd	Ctrl, comp	HS, comp
N	53	53	53	52	51
N+S	54	50	52	57	59
S	35	35	35	36	33

Table S2.2: Sample sizes per cell for analysis of female thermal sensitivity of fertility

Regime	control	HS
N	51	50
N+S	54	53
S	35	35

Table S2.3: Sample sizes per cell for analysis of transgenerational effects

Parent sex	female		male	
Parent HS	control	HS	control	HS
F1, control	46	43	45	44
F1, female HS	49	47	38	49
F1, male HS	43	52	39	47

Supplementary material S5:

Effect of multiple mating in the S regime.

The strongest decline in fertility was observed following the second mating of heat shocked S males. We here show that S males do not show such a decline in fertility if kept under benign conditions, and that this effect can be assigned to the heat shock treatment. We ran a follow-up experiment in which we allowed males (N=49) from the S regime kept at benign conditions to mate two times with 6 hours in between matings, as was done for males that had been heat shocked in the main experiment.

We first analysed differences in fertility between first and second matings of S males kept under benign conditions (i.e., the data collected during the follow-up experiment). We then also included data from the main experiment on fertility of isolated control males (first mating) and from the second mating of isolated heat shocked males. The term [experiment] represents from which experiment the males were taken and the interaction term [mating:experiment] tests the statistical significance of heat shock in the second mating.

Effects of mating number in males kept in benign conditions (Follow-up):
glm(offspring~ mating*line , family ="quasipoisson", data = followUp[follow$experiment == “followUp”,])

Analysis of Deviance Table (Type III tests)			
	X^2	df	p-value
mating	2.41	1	0.122
line	0.08	1	0.77
mating:line	0.73	1	0.3917

Effects of mating number in males exposed to heat shock (main experiment):
glm(offspring~ mating*line , family ="quasipoisson", data = followUp[follow$experiment == “Main”,])

Analysis of Deviance Table (Type III tests)			
	X^2	df	p-value
mating	9.16	1	**0.002****
line	0.02	1	0.88
mating:line	0.24	1	0.62

Effects of heat shock on fertility decline:

glm(offspring~ mating*experiment*line , family ="quasipoisson", data = follow2)

Analysis of Deviance Table (Type III tests)			
	X^2	df	p-value
mating	2.47	1	0.12
experiment	1.10	1	0.29
line	0.12	1	0.73
mating:experiment	5.77	1	**0.016***
mating:line	0.91	1	0.34
experiment:line	0.02	1	0.88

Supplementary figure S6:

Experimental design: transgenerational experiment

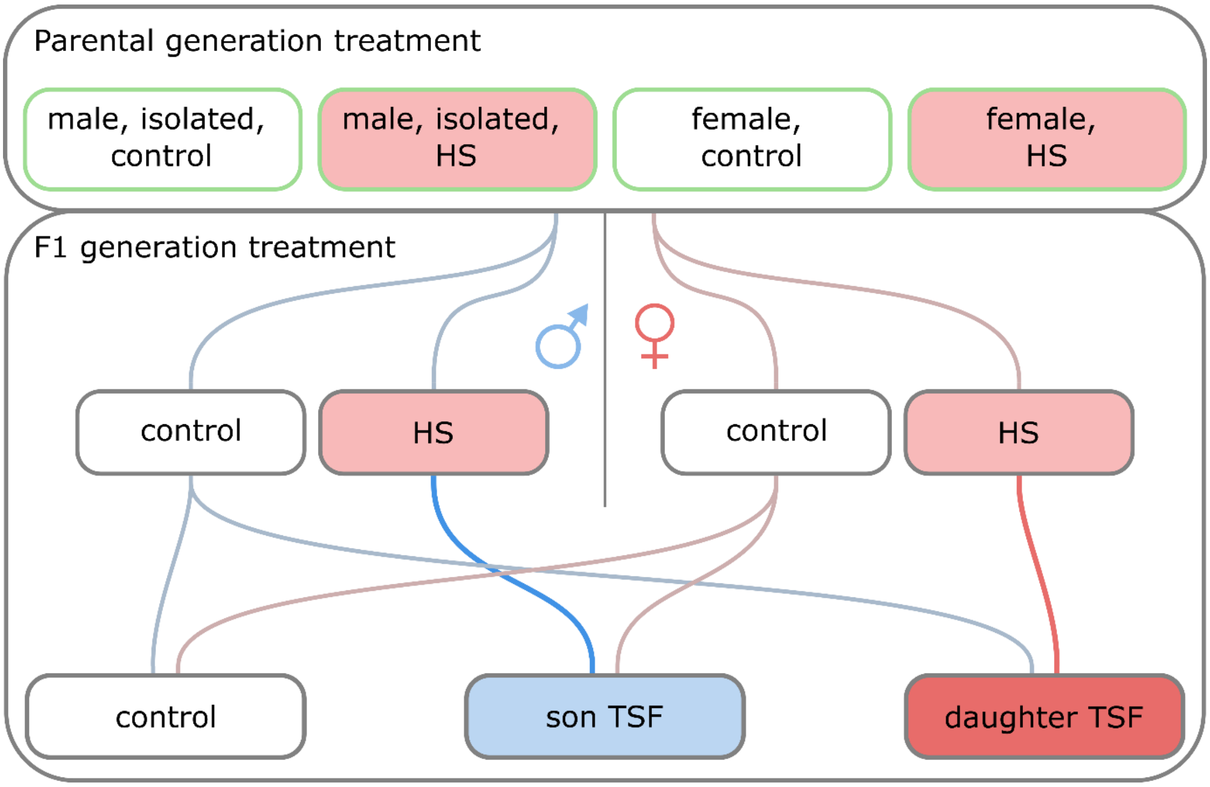

The experimental design used to assess transgenerational effects in offspring of heat shock in male and female parents. Focal parents were from N+S lines and were either control individuals or had been exposed to heat shock in the first generation of the experiment. Maternal and paternal transgenerational effects of the parental heat shock treatment was assessed by measuring the fertility of F1 offspring mating pairs. To assess whether the parental heat shock influenced the TSF of F1 sons and daughters, we also applied heat shock to the F1 generation in a sex-specific manner before assaying fertility. All offspring crosses were performed within line replicate and parental treatment group, while avoiding sib-mating. We used the same experimental procedure as in the main experiment for the heat treatment and fitness assays.

Supplementary table S7:

Full model specifications and output of the MCMCglmm used to analyse thermal sensitivity of fertility of the first and second mating of males kept in isolation prior to the heat shock application.

For this analysis heat shock treatment and mating number were combined in one factor with three levels (control, heat shock first mating (1h after heat shock), and heat shock second mating (7h after heat shock)).

Data: Male TSF data, excluding males that were exposed to socio-sexual competition (see S2.1, excluding "Ctrl, comp" and "HS, comp").

prior_male = list(R = list(V = diag(9), nu = 10^-6), G = list(G1 = list(V = 1, nu = 10^-6), G2 = list(V = 1, nu = 10^-6), G3 = list(V = 1, nu = 10^-6), G4 = list(V = 1, nu = 10^-6), G5 = list(V = 1, nu = 10^-6)))

MCMCglmm(offspring ~ regime*treatment, random = ~line + treatment:line + block + treatment:block + ID, rcov = ~ idh(treatment:regime):units, data = F1males[F1males$competition == "No",], family = "poisson", prior = prior_male, nitt=2200000, slice=TRUE, burnin=200000, thin=2000, verbose = FALSE)

Fixed effects:				
	posterior mean	lower 95% CI	upper 95% CI	p_{MCMC}
regimeN - Control (Intercept)	4.169	4.051	4.293	**<0.001*** **
regimeS	0.063	-0.063	0.173	0.244
regimeNS	0.084	-0.044	0.198	0.178
treatmentHS1	-0.134	-0.348	0.068	0.230
treatmentHS2	-0.064	-0.204	0.075	0.346
regimeS:treatmentHS1	-0.069	-0.343	0.207	0.618
regimeNS:treatmentHS1	0.146	-0.064	0.371	0.198
regimeS:treatmentHS2	-0.260	-0.489	-0.037	**0.014***
regimeNS:treatmentHS2	-0.01	-0.152	0.102	0.756

Random effects:				
	posterior mean	lower 95% CI	upper 95% CI	eff.samp
line	0.0016	2.69E-07	0.0070	731.5
treatment:line	0.0002	1.12E-07	0.0010	740.7
block	0.0028	2.08E-07	0.0120	909.3
treatment:block	0.0075	7.14E-07	0.0184	510.1
ID	0.0064	1.10E-07	0.0174	79.06

Supplementary table S8:

Full model specifications and output of the MCMCglmm used to analyse effects of male-male competition on thermal sensitivity of fertility of the first ejaculate.

Data: Male TSF data, only including data on first ejaculates of isolated males (see S2.1, excluding "HS, iso, 2nd").

Prior_comp = list(R = list(V = diag(12), nu = 10ˆ-6), G = list(G1 = list(V = 1, nu = 10ˆ-6), G2 = list(V = 1, nu = 10ˆ-6), G3 = list(V = 1, nu = 10ˆ-6), G4 = list(V = 1, nu = 10ˆ-6), G5 = list(V = 1, nu = 10ˆ-6), G5 = list(V = 1, nu = 10ˆ-6)))

MCMCglmm(offspring ~ regime*HS*competition, random = ~line + HS:line + block + HS:line:competition + HS:block + line:competition, rcov = ~ idh(HS:competition:regime):units, data = F1males[F1males$treatment != "IsoHS2",], family = "poisson", prior = prior_comp, nitt=2200000, slice=TRUE, burnin=200000, thin=2000, verbose = FALSE)

Excluding males that failed to mate:

Fixed effects:				
	posterior mean	lower 95% CI	upper 95% CI	p_{MCMC}
regimeN (Intercept)	4.167	4.045	4.295	**<0.001*** **
regimeS	0.069	-0.076	0.206	0.308
regimeNS	0.086	-0.044	0.241	0.194
HSYes	-0.130	-0.338	0.061	0.207
competitionYes	-0.019	-0.125	0.087	0.692
regimeS:HSYes	-0.080	-0.366	0.201	0.569
regimeNS:HSYes	0.142	-0.091	0.349	0.204
regimeS:competitionYes	-0.076	-0.208	0.062	0.231
regimeNS:competitionYes	-0.170	-0.360	0.031	**0.083**
HSYes:socioYes	0.111	-0.097	0.301	0.261
regimeS:HSYes:competitionYes	0.097	-0.222	0.372	0.511
regimeNS:HSYes:competitionYes	-0.020	-0.297	0.231	0.895

Random effects:				
	posterior mean	lower 95% CI	upper 95% CI	eff.samp
line	0.0015	1.49E-07	0.0066	1090
HS:line	0.0011	1.13E-07	0.0052	797.8
block	0.0031	1.46E-07	0.0115	512.2
HS:line:competition	0.0002	8.17E-08	0.0014	556.1
HS:block	0.0045	1.43E-07	0.0116	423
line:socio	0.0004	1.01E-07	0.0018	852

Including males that failed to mate as couples with offspring equal to zero:

Fixed effects:				
	posterior mean	lower 95% CI	upper 95% CI	p_{MCMC}
regimeN (Intercept)	4.175	3.984	4.368	**<0.001*** **
regimeS	0.065	-0.067	0.182	0.270
regimeNS	0.088	-0.035	0.226	0.172
HSYes	-0.203	-0.524	0.103	0.187
socioYes	-0.017	-0.131	0.077	0.720
regimeS:HSYes	-0.001	-0.309	0.285	0.983
regimeNS:HSYes	0.116	-0.186	0.353	0.4393
regimeS:socioYes	-0.078	-0.198	0.054	0.244
regimeNS:socioYes	-0.180	-0.385	0.001	**0.058**
HSYes:socioYes	0.009	-0.284	0.303	0.934
regimeS:HSYes:socioYes	0.058	-0.297	0.454	0.761
regimeNS:HSYes:socioYes	0.133	-0.219	0.519	0.488

Random effects:				
	posterior mean	lower 95% CI	upper 95% CI	eff.samp
line	0.0006	1.19E-07	0.0029	627.6
HS:line	0.0012	1.80E-07	0.0061	977.9
block	0.0059	1.23E-07	0.0287	1090
HS:line:socio	0.0003	1.97E-07	0.0017	828.1
HS:block	0.0346	0.005	0.0743	1090
line:socio	0.0003	9.11E-08	0.0012	536.7

Supplementary table S9:

Full model specifications and output of the MCMCglmm used to analyse thermal sensitivity of fertility of females.

Data: Female TSF data (see S2.2, all data).

Prior_female = list(R = list(V = diag(6), nu = 10ˆ-6), G = list(G1 = list(V = 1, nu = 10ˆ-6), G2 = list(V = 1, nu = 10ˆ-6), G3 = list(V = 1, nu = 10ˆ-6), G4 = list(V = 1, nu = 10ˆ-6)))

MCMCglmm(offspring ~ regime*HS, random = ~line + HS:line + block + HS:block, rcov = ~ idh(regime:HS):units, data = females, family = "poisson", prior = prior_female, nitt=2200000, slice=TRUE, burnin=200000, thin=2000, verbose = FALSE, pr = TRUE)

Fixed effects:				
	posterior mean	lower 95% CI	upper 95% CI	p_{MCMC}
regimeN (Intercept)	4.0540	3.735	4.345	**<0.001*** **
regimeS	0.366	0.044	0.721	**0.032***
regimeNS	0.337	-0.0061	0.666	**0.0456**
HSYes	0.226	-0.156	0.517	0.196
regimeS:HSYes	-0.477	-0.907	-0.0001	**0.040***
regimeNS:HSYes	-0.300	-0.677	0.097	0.142

Random effects:				
	posterior mean	lower 95% CI	upper 95% CI	eff.samp
line	0.0051	1.22e-07	0.0237	1000
HS:line	0.0023	1.84e-07	0.0129	672.1
block	0.0012	1.74e-07	0.0066	1000
HS:block	0.0011	2.41e-07	0.0059	1000

Supplementary figure S10:

Figure of effects of socio-sexual interactions and heat shock equivalent to figure 3 in the main text but including males that failed to mate as zero offspring.

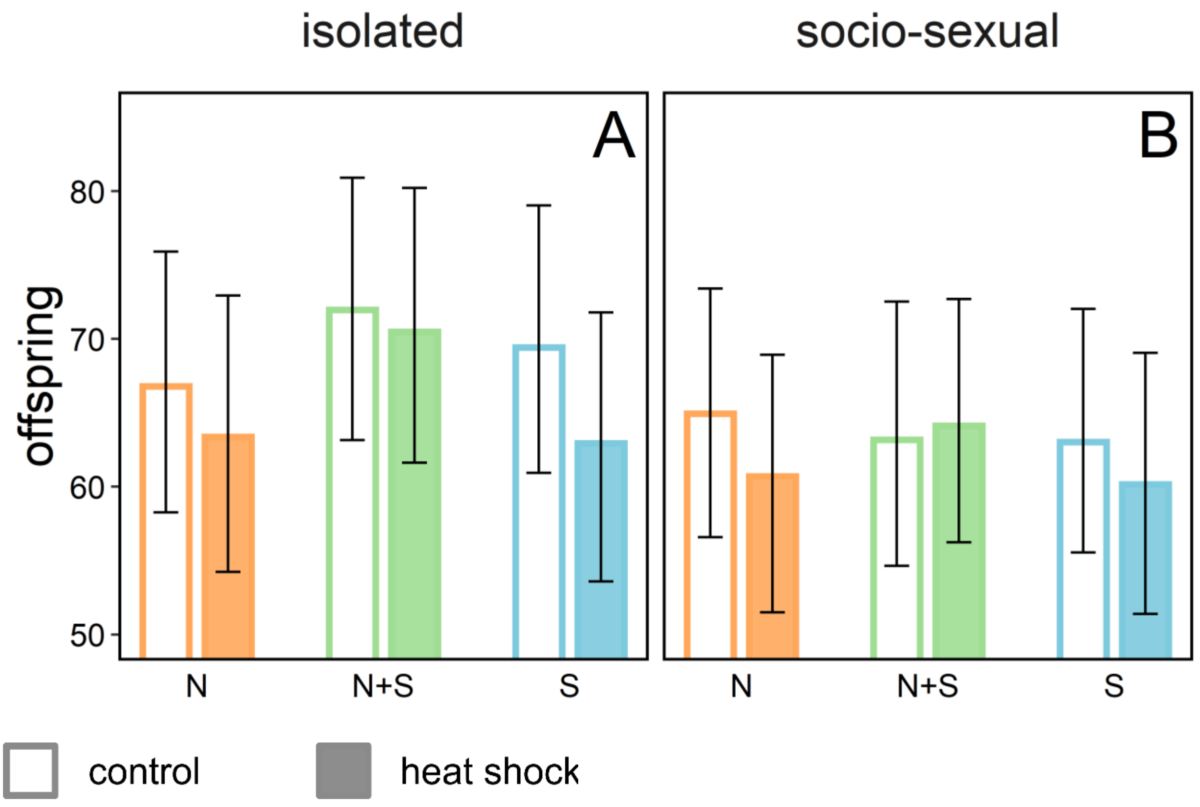

The effect of male-male interactions on male fertility. Fertility of couples from the N (orange), N+S (green) and S (blue) regime. Focal males were either kept at benign conditions (open bars) or exposed to heat shock (closed bars) and were either kept isolated or in groups of three prior to heat shock and mating. Bars represent posterior means and whiskers 95% credible intervals.

Supplementary table S11:

Full model specifications and output of the MCMCglmm used to analyse transgenerational effects of a heat shock on offspring quality and thermal sensitivity of fertility.

This analysis includes the sex of the focal parent (originating from the selection line, while the mating partner originated from the ancestral population) (male/female), parental treatment (control/heat shock), and the treatment of the offspring couple (male heat shock/female heat shock/control).

Data: F2 offspring data (see S2.3, all data).

prior_transgen = list(R = list(V = diag(3), nu = 10^-6), G = list(G1 = list(V = 1, nu = 10^-6), G2 = list(V = 1, nu = 10^-6), G3 = list(V = 1, nu = 10^-6), G4 = list(V = 1, nu = 10^-6)))

MCMCglmm(offspring ~ P.sex*P.treatment*F1.treatment + line, random = ~ block + P.sex:block + P.treatment:block + P.sex:P.treatment:block, rcov = ~ idh(F1.treatment):units, data = F2data, family = "poisson", prior = priorF2.1, nitt=2200000, slice=TRUE, burnin=200000, thin=2000, verbose = FALSE)

Fixed effects:				
	posterior mean	lower 95% CI	upper 95% CI	p_{MCMC}
P.sexF (Intercept)	4.280	4.208	4.346	**<0.001*** **
P.sexM	0.104	0.011	0.191	**0.026***
P.treatmentHS	0.016	-0.057	0.086	0.652
F1.treatmentF	-0.008	-0.082	0.064	0.804
F1.treatmentM	-0.056	-0.144	0.037	0.238
P.sexM:P.treatmentHS	-0.123	-0.220	-0.024	**0.016***
P.sexM:F1.treatmentF	-0.039	-0.144	0.072	0.462
P.sexM:F1.treatmentM	0.029	-0.114	0.151	0.640
P.treatmentHS:F1.treatmentF	-0.026	-0.129	0.080	0.604
P.treatmentHS:F1.treatmentM	-0.027	-0.145	0.098	0.688
P.sexM:P.treatmentHS:F1.treatmentF	0.081	-0.064	0.233	0.302
P.sexM:P.treatmentHS:F1.treatment M	-0.016	-0.184	0.171	0.832
Line 2	0.092	0.054	0.134	**<0.001*** **
Line 3	0.001	-0.044	0.042	0.932

Random effects:				
	posterior mean	lower 95% CI	upper 95% CI	eff.samp
block	0.0252	1.44E-07	0.0286	1000
P.sex:block	0.0014	1.11E-07	0.0042	1039
P.treatment:block	0.0006	1.44E-07	0.0028	1000
P.sex:P.treatment:block	0.0004	1.01E-07	0.0018	870.7

Supplementary note S12:

Exploration of the mechanistic links between investment in sperm competition and thermal sensitivity of male fertility.

To explore possible mechanistic links between the evolution of sexually selected postcopulatory reproductive traits and male TSF, we made use of previously published data from the same eight replicate lines as used here on sperm production (males were mated three times within 90 minutes to deplete sperm storage, 25 hours later males were allowed to mate again, sperm production refers to the increase in sperm number per ejaculate between the third and fourth mating), following 29 generations of experimental evolution (Extended data Fig. 5 in Baur & Berger 2020), and postcopulatory reproductive success in form of sperm defence (P1, i.e., the focal male is the first of two males to mate with the female) and sperm offense (P2, i.e., the focal male is second of two males to mate with the female), following 51 generations of experimental evolution (Fig. 1 in Koppik et al. 2022). We then estimated genetic correlations between these traits and the fertility reduction induced by heat shock (i.e., the TSF) in the second mating, based on line means. P1 and P2 were logit-transformed before analysis, as these traits are proportions ranging between 0 and 1.

We found a strong and statistically significant correlation between male TSF (assayed in the second mating) and previously reported estimates of male success in sperm offense, P2 ($r = 0.89$, $p = 0.003$), but not for sperm defence, P1 ($r = 0.39$, $p = 0.34$) nor sperm production ($r = 0.03$, $p = 0.95$), implying that improvement in a male's sperm offense is associated with increased sensitivity to thermal stress (Fig. S12).

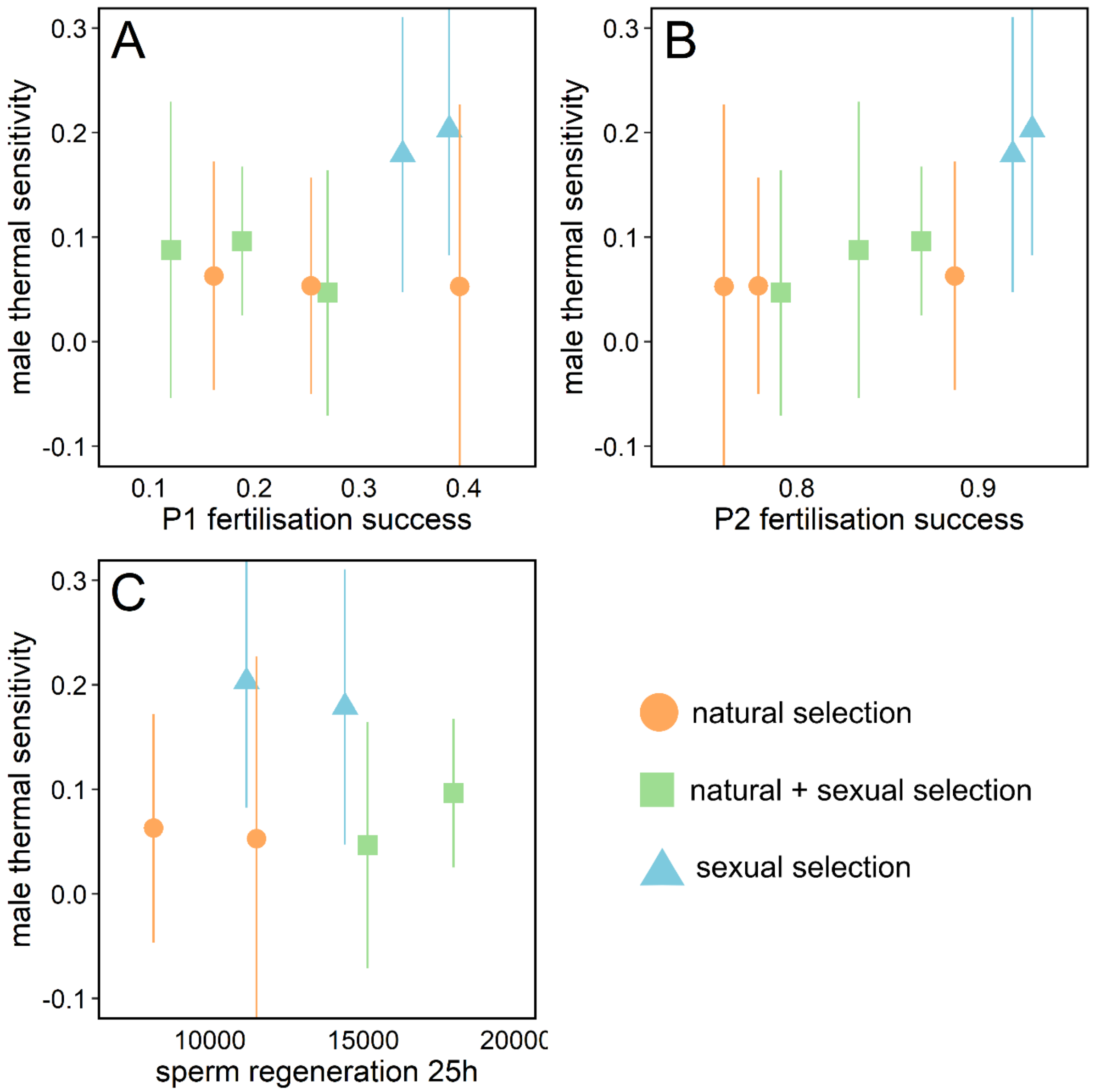

Figure S12: Correlations between male TSF and postcopulatory traits: (a) sperm defence (P1 fertilisation success), (b) sperm offense (P2 fertilisation success) (P1 & P2 data from Koppik et al., 2022), and (c) sperm regeneration (measured as the number of sperm transferred in mating, 25 hours after sperm depletion; data from Baur & Berger, 2020). P1 and P2 were logit-transformed before analysis. Individual data points represent line means for N (orange circles), N + S (green squares), and S lines (blue triangles). Whiskers represent 95% Bayesian credible intervals. Note that the axes of some panels have been adjusted for better illustration, which cuts off the error bars in some instances.
